# Toward accurate species‐level metabarcoding of arthropod communities from the tropical forest canopy

**DOI:** 10.1002/ece3.4839

**Published:** 2019-03-04

**Authors:** Thomas J. Creedy, Wui Shen Ng, Alfried P. Vogler

**Affiliations:** ^1^ Department of Life Sciences Natural History Museum London UK; ^2^ Department of Life Sciences Imperial College London Silwood Park Campus Ascot UK

**Keywords:** arthropods, body size, compositionality, metabarcoding, sequencing bias, species diversity, taxon composition

## Abstract

1. Metabarcoding of arthropod communities can be used for assessing species diversity in tropical forests but the methodology requires validation for accurate and repeatable species occurrences in complex mixtures. This study investigates how the composition of ecological samples affects the accuracy of species recovery.
2. Starting with field‐collected bulk samples from the tropical canopy, the recovery of specimens was tested for subsets of different body sizes and major taxa, by assembling these subsets into increasingly complex composite pools. After metabarcoding, we track whether richness, diversity, and most importantly composition of any size class or taxonomic subset are affected by the presence of other subsets in the mixture.
3. Operational taxonomic units (OTUs) greatly exceeded the number of morphospecies in most taxa, even under very stringent sequencing read filtering. There was no significant effect on the recovered OTU richness of small and medium‐sized arthropods when metabarcoded alongside larger arthropods, despite substantial biomass differences in the mixture. The recovery of taxonomic subsets was not generally influenced by the presence of other taxa, although with some exceptions likely due to primer mismatches. Considerable compositional variation within size and taxon‐based subcommunities was evident resulting in high beta‐diversity among samples from within a single tree canopy, but this beta‐diversity was not affected by experimental manipulation.
4. We conclude that OTU recovery in complex arthropod communities, with sufficient sequencing depth and within reasonable size ranges, is not skewed by variable biomass of the constituent species. This could remove the need for time‐intensive manual sorting prior to metabarcoding. However, there remains a chance of taxonomic bias, which may be primer‐dependent. There will never be a panacea primer; instead, metabarcoding studies should carefully consider whether the aim is broadscale turnover, in which case these biases may not be important, or species lists, in which case separate PCRs and sequencing might be necessary. OTU number inflation remains an issue in metabarcoding and requires bioinformatic development, particularly in read filtering and OTU clustering, and/or greater use of species‐identifying sequences generated outside of bulk sequencing.

## 1 INTRODUCTION

The great diversity of arthropods, challenging to study with traditional taxonomic methods, is increasingly being investigated with metabarcoding, that is, the PCR amplification and next‐generation sequencing of bulk samples obtained from mass trapping. For highly diverse arthropods of the tropical rainforest canopy, metabarcoding may be suitable to answer fundamental questions about the magnitude and distribution of species richness within and among different host tree species, to improve existing estimates of total species richness on Earth (Erwin, 1982; Hamilton et al., 2010; Ødegaard, 2000) and to better understand the ecology of this understudied community (Nakamura et al., 2017). Metabarcoding is already widely used in studies of arthropods, such as the pioneering work on Lepidoptera in (sub)tropical forests (Ji et al., 2013; Yu et al., 2012) and soil and aquatic macroinvertebrates (Arribas, Andújar, Hopkins, Shepherd, & Vogler, 2016; Fonseca et al., 2014; Macher et al., 2016). These studies have shown that metabarcoding is a highly suitable method for assessing total richness and species turnover for many topics from evolutionary biology to environmental monitoring (Andújar et al., 2018; Elbrecht, Vamos, Meissner, Aroviita, & Leese, 2017; Gibson et al., 2015; Hajibabaei, Baird, Fahner, Beiko, & Golding, 2016).

While metabarcoding is showing great promise, the precise methodology remains in flux. Studies of mixed species assemblages have recovered a relatively high number of operational taxonomic units (OTUs: clusters of sequence reads that aim to be equivalent to biological species), even when invisible with macroscopic methods (Arribas et al., 2016) or solely detected as environmental traces (Fonseca et al., 2014). This raises the possibility of inflated OTU numbers due to artifacts of the amplification process, including the formation of chimerical sequences, sample contamination, and the amplification of pseudogenes, among others. Conversely, other factors might lead to an underestimate of species numbers, for example, if primers skew PCR success or if reads are dominated by a few species in the specimen mixture due to high biomass. Recent studies have attempted validation of metabarcoding for arthropods, frequently by constructing mock communities to test the effects of primer choice (Elbrecht & Leese, 2015; Krehenwinkel et al., 2017), taxonomic composition (Krehenwinkel et al., 2018; Morinière et al., 2016), and amount of input DNA (Elbrecht et al., 2017; Krehenwinkel et al., 2017). This work has resulted in specific recommendations for improved methodologies, but it is limited by the focus on particular species and the low complexity of artificial communities.

In moving from contrived specimen mixtures to the analysis of real‐world samples, particularly in highly diverse communities of never‐before‐sequenced species as those from tropical forests, we are faced with several challenges in molecular methodology and bioinformatics. Ideally, to characterize large numbers of samples, we would use trap‐collected samples without time‐consuming prior sorting and instead apply metabarcoding to the full mixture regardless of taxonomic composition, relative abundance, or biomass of species present. However, as the complexity of the sample increases, differences in the amount of tissue between species (Elbrecht & Leese, 2015; Krehenwinkel et al., 2017) and primer skew (Arribas et al., 2016; Elbrecht & Leese, 2015; Krehenwinkel et al., 2017) may favor a few large‐bodied or abundant species and particular taxonomic groups, while negatively affecting the detection of others. The accuracy of metabarcoding would thus depend on the wider composition of the pool, as the various templates compete in different context of other templates, which also differ in their numbers.

Ultimately, while studies of mock communities can test the parameters that most strongly affect the efficiency and accuracy of metabarcoding, and provide avenues for mitigating against these confounding parameters, they are not suited to determine whether a given real community of unknown composition is characterized consistently. To make these compositional assessments more realistic, we take an approach that uses several samples collected under very similar conditions, but whose individual composition is unknown, to evaluate the error due to the structure of each sample. Specifically, we assess (a) the degree to which specimens of each of four body size classes are recovered if assessed on their own or in the context of larger‐bodied species, and (b) how the presence or absence of particular taxonomic groups (orders of arthropods) in a metabarcoding mixture might affect the recovery of other groups. We examine multiple samples, measuring both the recovery OTU richness/alpha‐diversity and the consistency of recovery of between‐sample beta‐diversity, utilizing both incidence (presence–absence)‐ and abundance (read numbers)‐based metrics.

## 2 MATERIALS AND METHODS

Samples were collected in Cusuco National Park, Cortés, Honduras, during a single 5% cypermethrin canopy fogging occasion of an individual *Liquidambar styracaflua* (Saxifragales: Altingiaceae) tree using circular 1 m^2^ trays suspended in the canopy at approximately 30 m above ground at very short distances from each other. Specimens collected in each tray formed a “tray sample.” Samples were stored in 100% ethanol and sorted to major taxa and/or size classes in the laboratory. In total, we used 13 tray samples from this fogging event, each representing natural communities sampled in a highly uniform way; thus, differences in composition were only due to the uneven distribution of species and stochastic differences in collecting success within a single tree canopy.

Size sorting was performed according to approximate body size, as measured under a dissecting scope. Four size classes were established, corresponding to cross‐sectional areas (body length × width) of 1–3 mm^2^, 3–9 mm^2^, 9–26 mm^2^, and 26–75 mm^2^, referred to as small, medium, large, and extra‐large, respectively. The number and boundaries of these size classes were established based on morphometric measurements of Coleoptera morphospecies, such that the mean volume of each size class was a constant multiple of the next smallest size class. Specimens smaller than 1 mm^2^ in cross‐sectional area (the smallest area that could be measured accurately) were included in the small class; for specimens larger than 75 mm^2^, tissue was sampled from the specimen and the tissue size determined its size class placement. The vast majority of specimens fell in the range 1–75 mm^2^. All sorting was performed under a microscope, and no attached external parasites or phoronts were observed – it is likely that any such arthropods were detached from their hosts upon death or during transport and manipulation of the samples, and thus would be properly categorized. Internal parasites/parasitoids could not be feasibly identified or separated from hosts, and this may cause bias whereby physically smaller arthropods were actually included in larger classes along with their hosts. Taxonomic sorting entailed the placement of specimens into one of eight “classes” of arthropods (usually orders), which included the vast majority of individual arthropods obtained. Formicidae were separated from the other Hymenoptera due to pilot data suggesting a high likelihood of primer mismatch (Supporting information Table S2).

### 2.1 DNA extractions, composite pool construction, and sequencing

**Figure 1:**
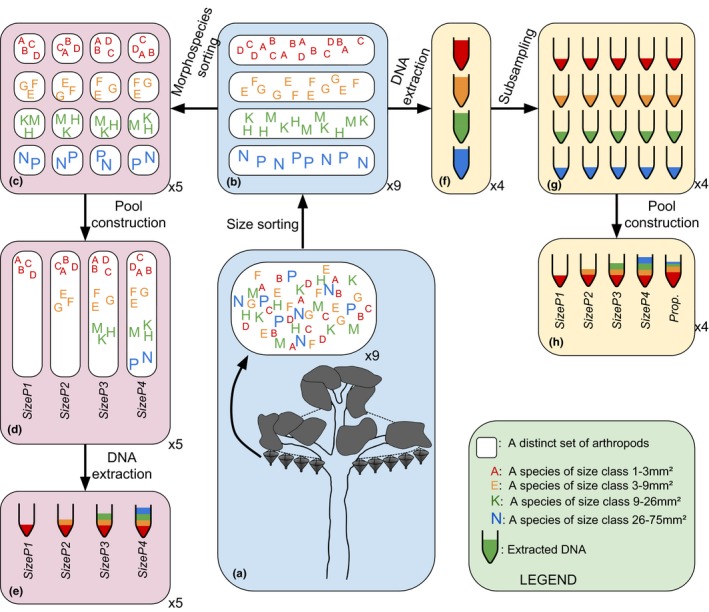
Diagram showing construction of size pools, both the Specimen method (a–e, blue and pink backgrounds) and the DNA method (a–b,f–h, blue and yellow backgrounds). Colored letters denote individual arthropods, each different letter a different morphospecies. Letter color and size denote size class, white boxes distinct samples, as shown in the legend. Tube cartoons denote distinct DNA samples, with colors denoting size class(es) of contents. For Specimen method size pool construction (blue, pink), arthropods from raw mixed tray samples (a) were sorted into size classes (b). Each size class was then sorted into subsamples of identical morphospecies and abundance composition (c). Four Size Pools (SizeP) were constructed from sets of these subsamples, such that each Size Pool contained a representative subsample of the size classes included (d). DNA was then extracted from each of these Size Pools (e). This process was carried out for five tray samples, resulting in 20 Size Pool samples for sequencing. For DNA size pool construction (blue, yellow), arthropods were also sorted to size class (a, b, as above). DNA was extracted from each size class (f), and each DNA extract was split into five equal parts (g). Size pools were constructed from sets of these equal parts as shown. For SP1–SP4, equal quantities of each size class were combined (h). For the Prop. composite pool, DNA was combined proportional to the inverse of the mean size of each size class, at a ratio of 64:16:4:1 from smallest to largest class. This process was carried out for 4 tray samples, resulting in 20 Size Pool samples for sequencing

Composite pools were created from sets of the size‐sorted or taxon‐sorted classes (“subcommunities”), as detailed in Figure 1, by sequentially adding more size classes (starting with the smallest) or by adding more taxonomic classes (starting with either Coleoptera, Formicidae, or Acari and adding one new taxon at a time). We did not equilibrate the concentration of DNA extract from different classes in order that combinations of extracts from a set of classes would be equivalent to an extraction of specimens from all of those classes together. To the four size pools (SizeP1–SizeP4, see Figure 1), a “proportional” pool (Prop.) was added by combining the four extractions in proportions inverse to their mean body sizes, that is, in ratios of 64:16:4:1 (small: medium: large: extra‐large) in order to normalize the effect of body size variation (proportional to specimen biomass, not considering their relative abundance in the natural sample; for an explanation, see Supporting information Appendix S1).

To extract DNA, dried specimen pools were suspended in 200–600 μl 1:9 Proteinase K and ATL and homogenized using a single 3 mm stainless‐steel ball bearing in a Qiagen TissueLyser II for 80–120 s at 30 Hz. After overnight lysis in a 56°C shaking incubator, samples were vortexed thoroughly and centrifuged at 3000 *g* for 3 min. The lysate supernatant was used for DNA extraction with Qiagen DNeasy Spin Columns. The resulting elutions were combined with others in specific ratios (see Figure 1). PCR was conducted on the DNA pools for 418 bp of the cytochrome oxidase subunit I (COI) barcode region using the primers Ill_B_F (Shokralla et al., 2015) and Fol_degen_rev (Yu et al., 2012) following the protocol of Arribas et al. (2016). Amplicons were sequenced on an Illumina MiSeq flow cell (2 × 300 bp paired‐end) after quality control, secondary PCR, and indexing with Nextera XT tags, at the sequencing facility, Natural History Museum London.

### 2.2 Bioinformatics and data processing

Bioinformatics processing was carried out using the NAPtime pipeline, a set of Perl scripts to wrap filtering and clustering software. NAPmerge carries out trimming, merging of paired‐end reads, and quality filtering/conversion using fastx_trimmer (Hannon Lab, 2012), PEAR (Zhang et al., 2014), and USEARCH fastq_filter (Edgar, 2010), respectively. A range of parameters was tested, but the final dataset used a PEAR‐q value of 26 and an fastq_filter expected error rate threshold of 1, also chosen by Arribas et al. (2016). NAPcluster carries out dereplication and size sorting of reads before denoising using USEARCH UNOISE (Edgar, 2016), clustering using USEARCH cluster_otus (Edgar, 2010) or swarm (Mahé, Rognes, Quince, de Vargas, & Dunthorn, 2015), and mapping reads to OTUs using USEARCH usearch_global (Edgar, 2010). NAPcluster also assigns OTUs a preliminary taxonomy based on parsing BLAST searches against the GenBank *nt* database. Only contigs with the expected length of 418 bp and unique sequences with >5 copies were retained. We considered five or fewer copies to be more likely to be sequencing errors rather than valid sequences. NAPcluster converts the output of usearch_global to a table of read numbers for each OTU in each sequenced pool (“composite community’). The output table for each composite community was rarefied to control for total read number and for the effect of dilution when comparing the OTUs within a particular size class or taxon class between composite pools of increasing complexity (Supporting information Table S1).

Each OTU was allocated to a size and/or taxon subcommunity based on its detection in the composite pools (Supporting information Figure S1). As the sequenced composite size pools were composed of only the small size class (named “SizeP1”) or the sequentially added three larger size classes (SizeP2 to SizeP4), only small‐sized OTUs could occur in all four composite communities, while the extra‐large OTUs should occur only in the composite communities of all size classes mixed together. Based on their incidence pattern, each OTU was assigned to one of the four size classes. An analogous approach was used for the assignment to taxon in the mixed pools of increasing taxonomic complexity. An OTU present in a single‐taxon pool can be assigned confidently to that taxon, while the first appearance in the sequential addition of other taxa determined the taxonomic assignment for other OTUs. Size class or taxon class assignment was then used to separate each composite community into a set of constituent subcommunities, in order to track each class‐assigned OTU in any particular sample or to determine the composition of an entire subcommunity.

### 2.3 Statistical analysis

For each subcommunity, the number of OTUs and the Shannon diversity was calculated. Shannon diversity was based on rarefied read numbers, and as such may be affected by stochastic variation in read number recovery proportional to true OTU abundances and so may not reflect true diversity. The significance of change in richness and diversity within each subcommunity between size‐based or taxonomy‐based composite communities was assessed fitting generalized linear mixed effects models (GLMM) using the lme4 package (Bates, Mächler, Bolker, & Walker, 2015) in R (R Core Team, 2018). Log‐log transformations were employed for the test of OTU richness, as the number of OTUs followed a Poisson distribution, and in both cases, the original tray sample ID was fitted as a random effect. Post hoc Tukey comparisons were calculated using the lsmeans package (Lenth, 2016). Where applicable, the number of OTUs in each set of taxa was compared with the number of morphospecies derived from parataxonomic sample sorting. The “proportional recovery” was calculated as (l+1)/(k+1), where *l* and *k* are the number of OTUs and morphospecies in a taxon, respectively.

To explore the effect of experimental community construction on observed beta‐diversity between the tray samples, multisample Jaccard and Bray–Curtis beta‐diversity indices were computed for each set of samples within each combination of size or taxon class, composite pool, and construction method. Finally, the combined read table was used to calculate the Jaccard and Bray–Curtis indices of total beta dissimilarity between size‐ or taxon‐based subcommunities. In both cases, the Jaccard index used only incidence (presence–absence) data while the Bray–Curtis index used read numbers as a proxy for abundance. Dissimilarity was visualized using ordination with nonmetric multidimensional scaling (NMDS; Kruskal, 1964, Supporting information Appendix S1), and the significance of dissimilarity/turnover in specific size or taxon subcommunities between increasingly complex composite pools was tested using GLMMs fitting the binomial distribution for proportion data and sample as a random effect. Analyses employed R (R Core Team, 2018) packages betapart (Baselga, Orme, Villeger, De Bortoli, & Leprieur, 2017) and vegan (Oksanen et al., 2017).

## 3 RESULTS

### 3.1 Sequencing data and OTU recovery

In total, we generated 80 metabarcode libraries with 3.9 million reads after pair merging and quality filtering, comprising 43,000 unique contigs of 418 bp in length and with >5 copies. After denoising and chimera filtering, these were reduced to 1,800 unique sequences. A wide range of clustering methods were applied, of which clustering with usearch_global version 9.2 at a 3% threshold was considered the most appropriate setting (Supporting information Figure S4), producing 913 OTUs across the entire dataset.

Across a set of sequenced pools for which parataxonomic data was recorded, the number of morphospecies was compared with the number of OTUs preliminarily classified into a set of 12 taxa. Samples were dominated by Coleoptera, followed by Araneae, Hemiptera, and Hymenoptera (Supporting information Figure S2a). Molecular OTU delimitation revealed significantly higher diversity than estimated from parataxonomy in many groups, with high ratios of molecular OTUs to morphospecies in particular in Coleoptera, Hemiptera, and Diptera (e.g., a ratio of 9.9 in the latter), although some samples also underestimated the morphological diversity (Supporting information Figure S2b). The mean proportional recovery of OTU to morphospecies across all taxa was 3.5 and significantly >1 (one‐sample *t* test, *t* = 9.2, 731 *df*,* p* < 0.001) for eight of the 12 taxa.

### 3.2 The effects of body size

**Figure 2:**
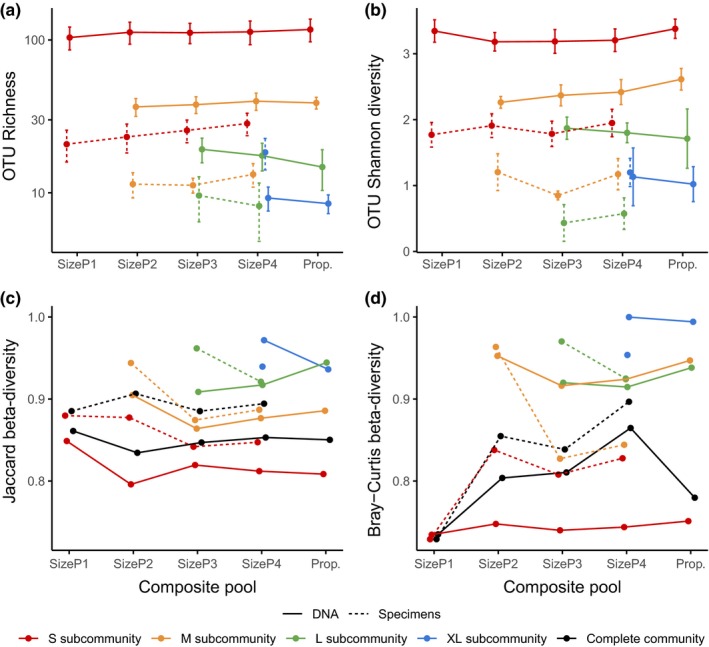
The recovery of OTUs and community turnover patterns for each of four size classes (colors), for each of five pool types (*x*‐axes), for two experimental community construction methods (line type, see Figure 1). Subplot (a) shows number of OTUs recovered, and subplot (b) shows Shannon diversity of OTUs taking into account read numbers. For (a and b), points and error bars show mean and standard error. Bottom plots report beta‐diversity ((c) Jaccard, using presence–absence only, (d) Bray–Curtis, using read numbers) between replicates (error bar range in top plots) for each pool type and construction method, for each separate size class subcommunity and for all subcommunities in the pool together. For example, four source samples were used to construct experimental pools by mixing DNA from different size classes in the laboratory (solid line). For the library where the small, medium, and large size classes were combined (SizeP3), we calculate beta‐diversity between the four small subcommunities from those replicates (red point), for the medium and large fractions as well (orange and green points), and for the complete community comprising all three subcommunities (black point)

Two experiments examined the effect of size composition of specimen mixtures on recovered OTU diversity. First, a tray sample was sorted into four size classes, DNA was extracted from each class separately, and the resulting extracts were used to create five pools, SizeP1–SizeP4 and Prop. This analysis was replicated on four separate tray samples (Figure 1, blue and pink panels). Metabarcoding produced a high number of small‐bodied OTUs and increasingly fewer OTUs for the other size classes. According to post hoc Tukey comparisons of GLMM fittings, there were no significant differences in mean OTU Richness or Shannon diversity between composite pools within each class within each construction method, taking account of variation between tray sample (Figure 2, solid lines, top panel).

A second experiment of sequential size class addition was performed with subcommunities consisting of defined morphospecies, conducted on five replicate tray samples. The selection required that species were present in sufficient numbers of individuals within each tray sample to include them in four exactly equal subsamples from which four size pools were produced (SizeP1–SizeP4; Figure 1, blue and yellow panels). These pooled communities were generally comprised of fewer OTUs than the pools of the first experiment (as only species with a minimum of four individuals were selected to be represented in each class), but again no significant differences in the number or diversity of OTUs within size classes were observed, that is, the addition of further size classes had little effect on the detection of the small‐sized OTUs (Figure 2, top panels, dashed lines).

For each experiment, we calculated beta‐diversity between equivalent subcommunities from the different replicates (tray samples) within each size class, composite pool, and construction method combination (Figure 2, bottom panels). The purpose of this was to examine the effect of pool composition and methodology on the apparent turnover between our real‐world samples. Average between‐sample (“real‐world”) turnover varied between different size classes and between experimental composite community construction method; however, the pattern of change in turnover with the addition of larger size classes was largely flat: Beta‐diversity remained consistent within each size class and construction method despite increasingly complex pool structure.

**Figure 3:**
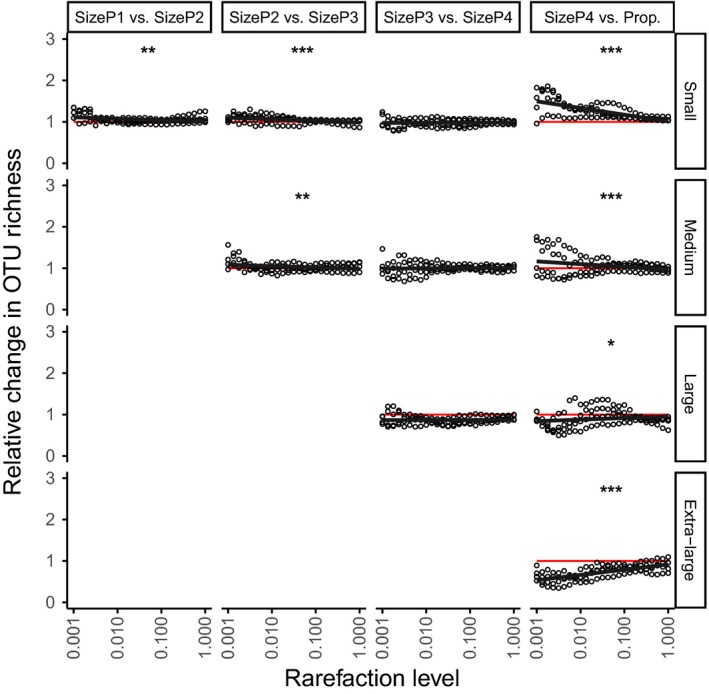
Relative change in number of OTUs recovered between pairs of composite pools (columns) within size classes (rows), over simulated variation in read depth, using only the data from the laboratory‐constructed samples. Read depth is represented by differing levels of rarefaction, representing increasingly lower read numbers. The *x*‐axis increases from 0.1% to 100% of the values used in rarefaction of this dataset for the main analyses (Supporting information Table S1). Columns of panels show pairwise comparisons between successive pairs of pools, for example, the rightmost panel compares the number of OTUs recovered in SizeP4 and Prop, split into the four different subcommunities based on size (rows). Comparisons are the proportional change in the number of OTUs: Where *T*_*C*_ is the number of OTUs in a less complex size class, and *T*_*C*+1_ is the number of OTUs in the next more complex, proportional change is calculated as $\left(T_{C+1}+1)/(T_{C}+1\right)$. Values above the red line show an increase in the number of OTUs recovered going from the less complex to the more complex experimental communities. Fitted lines are lmer fits controlling for variation between sample, and stars show significance of slope compared with 0 (*0.01 < *p* < 0.05, **0.001 < *p* < 0.01, ***p < 0.001)

Finally, we tested the degree to which these conclusions depend on the sequencing depth, using a range of lower rarefaction targets to simulate decreased sequencing intensity. We rarefied to between 0.001 and 1 times the lowest read number available. To reduce the effects of stochasticity in rarefaction, especially at low target values, we repeated rarefaction at each target total read number 20 times and averaged the resulting OTU read numbers. The effect of adding another size class on the recovery of OTU richness was compared directly between pairs of consecutively more complex size pools, for example, SizeP1 vs. SizeP2, SizeP2 vs. SizeP3, and so on up to SizeP4 vs. Prop. At lower sequencing depth, we might expect that adding a larger size class to a composite pool would have a greater effect on the difference in the observed OTU richness of a smaller size class subcommunity. However, this experiment showed no or very marginal change in OTU recovery differences between most adjacent size pools with stricter rarefaction (Figure 3). However, when comparing the two composite communities where all size classes were represented, but at different ratios, (SizeP4 vs. Prop.), higher levels of rarefaction (i.e., lower sequencing depth) generally resulted in a greater proportions of OTU changes. As “sequencing depth” decreased, the smallest size classes were recovered significantly better through proportional recombination of size classes in a pool (Prop.) compared with equal‐volume (SizeP4), while recovery of the largest size classes was significantly poorer for the same comparison.

### 3.3 The effects of taxonomic composition

**Figure 4:**
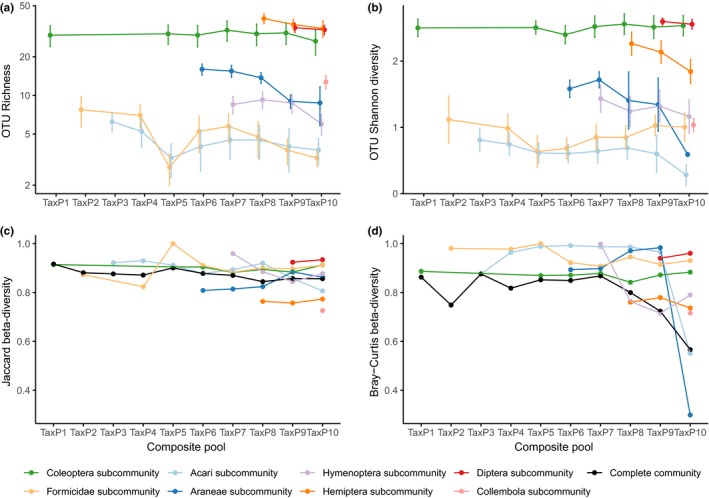
The recovery of OTUs and community turnover patterns for different taxa (colors), for each of 10 pool types (*x*‐axes). Subplot (a) shows number of OTUs recovered, and subplot (b) shows Shannon diversity of OTUs taking into account read numbers. For (a and b), points and error bars show mean and standard error. Note that where taxon point is absent for a pool type (e.g., there is no green Coleoptera point for TaxP2), this taxon was not included in this construction. Bottom plots report multisample beta‐diversity ((c) Jaccard, using presence–absence only, (d) Bray–Curtis, using read numbers) between replicates for each pool type, for each separate taxon subcommunity and for all subcommunities in the pool together. For example, four source samples were used to construct experimental pools. For the library where the Coleoptera, Acari, and Formicidae were combined (TaxP5), we calculate beta‐diversity between the four Coleoptera subcommunities from those replicates (green point), for the Acari and Formicidae fractions as well (light orange and light blue points), and for the complete community comprising all three subcommunities (black point)

**Figure 5:**
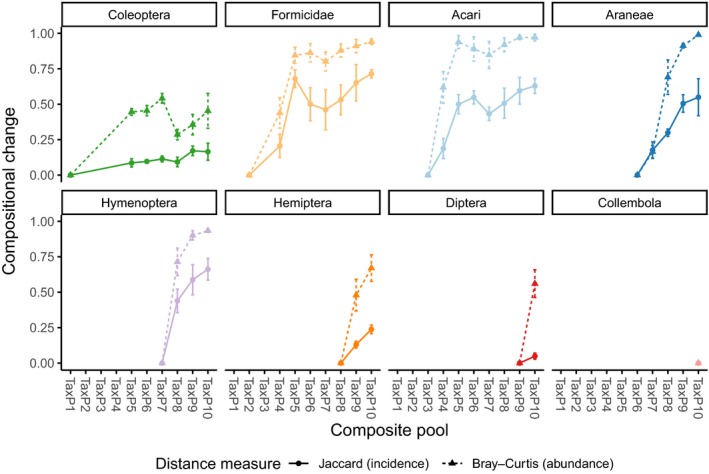
Plots of compositional dissimilarity between the least‐dilute subcommunity for a taxon and subsequent subcommunities as part of more complex experimental communities. Each panel is a different taxon, and the *x*‐axis is experimental pools in increasing order of complexity. *Y*‐axis shows compositional dissimilarity, with 0 = identical and 1 = completely dissimilar (no shared OTUs). Line types show dissimilarity measure: Jaccard (presence–absence only) or Bray–Curtis (read numbers). Points and error bars show mean and standard error. Note that the leftmost point in each plot is the reference subcommunity and therefore will always have a dissimilarity value of 0

The effects of taxonomic composition on metabarcoding success were tested with tray samples sorted into eight higher taxa of arthropods. Separate DNA extractions from each taxon were combined to create 10 composite taxonomic pools (TaxP1–10). For most taxa, there was a slight decline in the number of OTUs recovered in increasingly complex composite communities, despite controlling for the dilution effect as relative read numbers decrease (Supporting information Table S4). For most taxa, OTU richness and diversity were not significantly affected by any single addition of further taxa (Figure 4, top panels). However, there was a significant decline in both Acari and Formicidae OTU richness (but not diversity) with the introduction of Coleoptera, and a similarly significant decline in Araneae with the introduction of Diptera. Notably, the OTU richness of Formicidae and Acari recovered somewhat and then declined again, whereas the richness of Araneae did not recover (although there were fewer samples to explore this pattern). However, when looking at compositional similarity, only the Coleoptera showed evidence that the same OTUs were recovered consistently, irrespective of the composition of the wider sample. All others showed increasing discrepancies from the OTU composition under low complexity (Figure 5).

As with the size experiment, we calculated the multisample beta‐diversity between the subcommunities from different samples within each taxon class, composite pool, and construction method combination (Figure 4, bottom panels). There was substantially less variation in beta‐diversity between different taxonomic fractions compared with size‐based fractions; however, there was considerably greater variation within taxon classes over different composite pools, especially when considering abundance‐based beta‐diversity.

## 4 DISCUSSION

We sequenced various reconstructions of community samples to test the reliability of metabarcoding for arthropod community ecology, in the face of many possible biases. Attempting a real‐world application of metabarcoding, we used actual community samples, rather than widely employed artificial mock communities. The design involved separate DNA extraction on size‐ and taxon‐based subcommunities and tests their recovery when combined with various portions of the full community. With this two‐step process of in vitro deconstruction and in silico reassembly, we were able to assign otherwise‐anonymous OTUs to specific subcommunities and to accurately trace the detection of these OTUs between samples. The resulting data show that, in general, size classes and taxa remain compositionally consistent when processed together in various combinations with other components of the pool community.

We recover significantly more OTUs than input morphospecies. We invested substantial effort to determine the most appropriate similarity levels for OTU delimitation (see Supporting information Appendix S1) and found that to recover an equal ratio of OTUs to morphospecies required parameters well outside the current standard practice (Supporting information Figure S4). We settled on the 3% value, which remains an arbitrary choice but is in line, on average, with levels defining intra‐ and interspecific differentiation for grouping barcode data (e.g., BINS, Ratnasingham & Hebert, 2013). We attribute OTU inflation to several issues: unidentifiable non‐target sequences (e.g., pseudogenes), systematic differences in molecular species divergences, or inability to accurately differentiate morphospecies during sorting. OTU limits certainly affect the assessment of richness in metabarcoding, but we believe that in this study, OTU inflation is a consistent bias that does not confound our interpretations of within‐study diversity and compositional variation. However, the lack of validated taxon concepts for sequencing clusters remains a major limitation to reliable biodiversity estimates, in particular in the study of unknown faunas lacking external sequence reference libraries. Further bioinformatic development is required to improve filtering of non‐target sequence reads and perhaps to adapt OTU delimitation to taxonomic context. Currently, the ideal metabarcoding study should include controls of known taxonomic composition and could be greatly improved by an approach that defines at least a subset of OTUs independently of metabarcoding, through individual barcoding or even mitochondrial sequencing. The latter approach adds considerable value to metabarcoding ecology by enabling phylogenetic reconstruction, which can be followed by placement of unknown OTUs to a tree for improved identification and filtering.

### 4.1 Biases in species composition

Size differences did not greatly or consistently affect species recovery in metabarcoding, given a certain level of sequencing depth. Specifically, the number and diversity of OTUs in each size class did not greatly change whether sequenced individually or together with other size classes (Figure 2). We observe that even as the proportion of DNA contributed by a subcommunity becomes smaller, the recovery of OTUs and OTU alpha‐diversity is reliable. The assays are sensitive and realistic, as evidenced by the very similar patterns observed comparing subcommunities generated from all DNA in the tray sample or only from particular species selected for manual assembly, the latter of which shows a lower total number of OTUs recovered. In general, we see no effect on incidence‐based beta‐diversity with increasingly complex mixed pools (within size class and method), showing that sample composition does not affect our ability to observe study‐level community structure. However, variability in turnover increases when using abundance‐based beta‐diversity and with specimen‐based construction method. We attribute this to greater stochasticity driven by variation in OTU read numbers between sequenced libraries, and by fewer OTUs with the specimen‐based method. These results also allow us to be confident that observed turnover of the composite and subcommunities is valid “natural” stochastic compositional change, as turnover varies largely as part of experimental modification rather than methodological error.

The result of lower sequencing coverage is inconsistent. With low rarefaction targets (simulating low sequencing depth), we would expect that the smallest OTUs are more likely to be recovered more poorly when sequenced alongside larger sizes than when sequenced alone: Instead, the results show a significant slight opposite effect for both the small and medium size classes comparing between the three less complex composite communities (Figure 3). Inversely, comparing composite communities with all size classes combined at different ratios (SizeP4 vs. Prop.), we see the expected response: The recovery of small OTUs is improved and the recovery of large OTUs is suppressed by proportional combination of DNA from different size classes at low read levels, but this effect diminished with higher read numbers (= higher rarefaction targets).

We find clear variation in the recovery of OTU richness and diversity of certain taxon classes once other taxa were included in the metabarcoding (Figures 4 and 5). The composition of all taxon subcommunities substantially changes with the addition of other taxa, and this is exacerbated when using abundance‐based measures of dissimilarity, with both assemblage and relative read numbers being affected by increasing taxonomic complexity. Most taxa respond with a roughly linear rate of compositional dissimilarity with increased community complexity, with a shallower response in the Coleoptera and a steeper response in the hymenoptera, suggesting a primer affinity in the former and greater incompatibility in the latter. Between‐sample beta‐diversity is considerably more inconsistent over increasingly complex communities compared with the size‐based study. The introduction of other taxa clearly affects the ability of metabarcoding to successfully recover a consistently accurate representation of the community structure of individual taxa, and this may considerably affect the ability of a study to report accurate measures of study‐level structure such as beta‐diversity. This is particularly the case if read numbers are used in an abundance‐based assessment of compositionality: Where only presence–absence is employed, beta‐diversity between samples is broadly consistent over differing taxonomic compositions.

While the Arribas et al. (2016) primers successfully amplify a wide range of arthropod lineages, it does appear that binding affinity is unevenly biased toward some taxa and away from others, leading to inaccurate representation of relative taxon richness and composition in mixed metabarcoding samples. In particular, these results suggest the primers have a greater affinity for Coleoptera DNA, as recovery of OTU richness, diversity, and composition of this taxon is clearly affected less by the inclusion of other taxa compared with the other groups. The particular affinity of Coleoptera for this primer sequence is further evidenced by the effect on the recovery of OTU richness and composition of the Formicidae and Acari by the addition of beetles in a three‐way combination (TaxP5). Fortunately, this appears to be the only taxon that causes this effect, and the extent of the disruption is somewhat ameliorated by the dilution of the Coleoptera through further addition of other taxa. Notably, the greatest changes in OTU numbers and turnover (Figures 4 and 5) occurred in groups that generally appear to be composed of fairly small numbers of OTUs, which may be subject to stochastic effects of species detection in the PCRs (although we combined three replicates) and sequencing. The stringent read quality filtering, chimera removal, and OTU clustering prior to the analysis of turnover probably minimize artifactual OTUs arising in the mixed DNA, but the conservative handling of reads could also result in low detection success for certain OTUs. False negatives in part would be dependent on read depth, which was addressed by rarefaction to control for the dilution of reads by the addition of new taxa in the taxonomic addition experiment.

### 4.2 Measuring community structure

We calculated community structure indices based on both OTU presence–absence and read numbers. While abundance‐based metrics are better able to represent the composition of a true ecological community, we cannot be certain that the use of read numbers is truly representative of ecological abundance, especially in samples of mixed size and taxonomy where the relationship between read numbers of an OTU and species abundance will be affected by primer affinity, DNA quantity, and sequencing stochasticity. In both size and taxonomy experiments in the present study, there was little difference in the observed patterns between OTU richness and Shannon diversity: Including read numbers did not affect the conclusions drawn. However, there was greater variation in the observed pattern of beta‐diversity calculated from read numbers compared with beta‐diversity calculated from presence–absence alone, although patterns were generally consistent in direction. We are cautious about deriving firm conclusions from abundance‐based composition metrics; while the variation could be interpreted to point toward size or taxon biases in recovery, it could also be due to many sources of error in the metabarcoding processes.

### 4.3 Implications for studies of canopy arthropods

These data join a relatively small cohort of studies that examine the entirety of a high‐diversity tropical arthropod community at a species‐equivalent level, and the separation of different subsets of this community revealed interesting patterns that should inform similar future studies. Small‐bodied OTUs dominated the communities, in line with known molecular abundance spectra of tropical insects (Choo, Crampton‐Platt, & Vogler, 2017). The canopy fogging method employed here, using small collecting trays suspended high in the canopy, is particularly efficient for catching small‐bodied specimens that would be missed in larger, ground‐level sampling screens due to drifting of specimens, while larger specimens may be missed due to their ability to escape from the small‐scale canopy fogging. The smallest specimens of the community are frequently not captured or are understudied by morphological approaches. Metabarcoding thus may be crucial in understanding the important contribution these species make to species composition and turnover, without the bias introduced by traditional taxonomy. Furthermore, morphological studies are frequently limited in taxonomic breadth and may misrepresent the extent and pattern of turnover across the entire Arthropoda, which is overcome by metabarcoding. In addition, the magnitude of compositional change across the tray samples (Supporting information Figure S3 and Table S3) clearly varies between different subcommunities, which provide additional information for arthropod community ecology from comparing separate subcommunities (e.g., small bodied vs. large specimen) without the need for direct characterization of the species involved.

The largest arthropods used in this study had a cross‐sectional area of 75 mm^2^; this may be considered to limit the applicability of these findings to metabarcoding studies that include much larger individuals. However, metabarcoding is most useful for smaller‐bodied individuals, which make up a disproportionate part of the species and individuals in most terrestrial arthropod communities. The power‐based grouping system used in this study allowed comparison between arthropods that varied in size by up to 64‐fold and found no difference in or effects on recovery rate. With sufficient sequencing, this pattern may be expected to hold true for arthropods at least another size class larger (up to 220 mm^2^, a fourfold increase on the largest individuals used in this study); alternatively, the largest individuals are easily extracted from mixed pools and can be tissue‐subsampled to be included fairly in metabarcoding. As such, this caveat is relatively minor and these results likely apply to most arthropod metabarcoding studies.

### 4.4 Implications for metabarcoding arthropod communities

The findings have obvious practical implications: Should we sort by size, or by taxon, or both, prior to DNA extraction and metabarcoding? The great power of metabarcoding clearly derives from the ability to go from the trap catch directly to DNA analysis of species composition without elaborate (para)taxonomic steps. Our results suggest that in most cases, size sorting and biomass control are not necessary with sufficient sequencing depth; however, some degree of taxonomic sorting and the use of taxonomic‐based control samples may be beneficial, in particular to gain additional ecological information. Size sorting is much easier than any kind of taxonomic sorting, although even sorting to order level can be performed relatively rapidly, perhaps while also gathering other valuable information such as specimens counts.

The biases from taxonomic composition of samples would generally suggest that where feasible this kind of separation is desirable, at least in taxa known to either strongly affect or be strongly affected by other taxa, such as the Coleoptera for these primers. In addition, performing multiple PCRs in combination and separately may give a more accurate picture of the total species diversity, which in individual reactions may be missed. It appears the lack of detection of many species in a particular run is not primarily due to low read depth, although this could potentially be increased to optimize the detection of rare reads when applying highly stringent quality filtering protocols, as was done here. The decision to apply metabarcoding to particular subsets of a mass‐trapped sample ultimately depends on the required accuracy of the data. For many applications of species turnover and total diversity, the exact number is not important, as long as a similar error is introduced in all samples equally. However, when the experiments require great precision of species lists, presorting of specimens by taxon and potentially also by size may be helpful, and in fact, using different primers may further avoid the inadvertent omission of species. At the same time, the frequently very high number of OTUs obtained in some studies (Bista et al., 2017) could include false positives that can be eliminated by only scoring OTUs consistently obtained from multiple separate amplifications and sequencing, or through use of separately prepared barcode or genomic datasets.

### 4.5 Conclusions

As we start using metabarcoding for studying the great diversity of arthropods of the rainforest canopy, to reassess the long‐standing questions about species numbers, host specificity, and species turnover, the validation of the approach requires that these entities are equivalent to the Linnaean species or morphospecies of existing studies of tropical insect diversity. OTU clusters here were defined with stringent methods for sequence quality and cluster threshold, which allowed to trace each cluster across natural communities and artificial subcommunities derived from them, and thus to test the effect of potentially confounding parameters of species detection. It was important that natural communities from trapping efforts were used, making the scenarios as realistic as possible. The consistent recovery of particular OTUs within and between natural samples shows that metabarcoding may be more rigorous, consistent, and have greater utility than simple parataxonomic morphospecies delimitation and identification. Arthropod ecologists can thus be confident that metabarcoding can generate comprehensive, realistic, and accurate community data, in particular for small‐bodied taxa, even without controlling for body size or taxonomic composition of samples. The high quality of metabarcoding data thus can contribute to the global effort for generating sequence data of all species on Earth, in particular for poorly known, diverse ecosystems such as the tropical rainforest canopy.

## CONFLICT OF INTEREST

APV is a co‐founder and scientific advisor of NatureMetrics, a private company providing commercial services in DNA‐based monitoring. The authors declare that they have no other possible conflicts of interest.

## AUTHOR CONTRIBUTIONS

T.J.C. and A.P.V. conceived the research, T.J.C. and W.S.N. designed and implemented the methodology, T.J.C. undertook the analysis, T.J.C. and A.P.V. wrote the paper.

## DATA ACCESSIBILITY

DNA extracts are deposited in the Molecular Collections Facility, NMHUK. The OTU sequences, read table, and sample metadata are available in the Dryad repository, https://doi.org/10.5061/dryad.120f446. All Supporting information, tables and figures, and R scripts for analysis, will be uploaded as online Supporting Information Appendix S1.
