# Supplementary material for: Toward accurate species‐level metabarcoding of arthropod communities from the tropical forest canopy
Source: Ecol Evol. 2019 Mar 4;9(6):3105–16. doi: 10.1002/ece3.4839 (PMC6434547; doi:10.1002/ece3.4839)
Supplement: Supplementary file 1 [file ECE3-9-3105-s001.pdf]

Supplementary Materials: Towards accurate species-level metabarcoding of arthropod communities from the tropical forest canopy

Thomas J. Creedy[12*], Wui Shen Ng[12], and Alfried P. Vogler[12]

[1]Department of Life Sciences, Natural History Museum, Cromwell Road, London SW7 5BD, UK
[2]Department of Life Sciences, Imperial College London, Silwood Park Campus, Ascot SL5 7PY, UK

*Corresponding author thomas@tjcreedy.co.uk

1 Supplementary Methods

1.1 Rationale for pooling strategy

Here we present the logical basis for our treatment of DNA extract from different subsets of a community in order to ensure that the final pipeline controls, as far as possible, the bias due to biomass in the recovery of an OTU and thus the final community dataset is as representative a sample of the true community than one processed solely by traditional taxonomic methods. We also present why it is not necessary to control for concentration in the combination of DNA extracts from different size classes

Assuming that an OTU is an exact representation of a species, let us take M to be the amount of DNA of an OTU in mixed DNA extract, B to be the average biomass of an individual of that OTU/species, A to be the number of individuals of that OTU/species in the community and D to be the amount of DNA per unit biomass for the OTU. Therefore:

$$M = (B \times D \times) \times A \tag{1}$$

The concentration C of this hypothetical sample is simply:

$$C = \frac{M}{V} \tag{2}$$

$$\therefore M = CV \tag{3}$$

$$\therefore V = \frac{1}{C} \times M \tag{4}$$

$$\tag{5}$$

Where V is the volume of the extract. We shall return to this below.

Fundamentally, we can assume that the probability of recovery of an OTU $\mathsf{P}(R)$ in a single mixed DNA extract is proportional to the amount of DNA M of that OTU, i.e. a function of M and some unknown constant k:

$$\mathsf{P}(R_{OTU}) = kM \tag{6}$$

This is of course assuming no other OTU-related effects on probability of recovery, such as taxonomy, but we are forced to ignore this for these purposes.

Finally, the standard assumption of community ecology is that the probability of recovery of a species in a sample is directly proportional to the abundance of that species in the community, i.e.:

$$\mathsf{P}(R_{Species}) = cA \tag{7}$$

We can see by comparison of equations 6 and 7 that the probability of recovery of an OTU is not the same as the probability of recovery of a species. From equation 1, $\mathsf{P}(R_{OTU})$ can only equal $\mathsf{P}(R_{Species})$ if $cA = kM = k(B \times D \times A)$ i.e. if biomass and DNA density is constant between OTUs, which of course it is not.

To make $\mathsf{P}(R_{OTU})$ equal to $\mathsf{P}(R_{Species})$, we must modify the the amount of DNA from an OTU present in the pool, so let M' be the modified amount, such that:

$$M' = \frac{M}{B \times D} = A \tag{8}$$

The amount of DNA is only accessible via the volume of extract, however (equation 2), therefore

$$M = CV \tag{9}$$

$$V' = \frac{1}{C'} \times M' \tag{10}$$

$$\therefore V' = \frac{1}{C'} \times \frac{CV}{B \times D} \tag{11}$$

However, while the amount of DNA M and the volume V of the extract changes while extraction only a portion (i.e. to M' and V'), the concentration does not. Thus:

$$C' = C \tag{12}$$

$$\therefore V' = \frac{1}{C} \times \frac{CV}{B \times D} \tag{13}$$

$$\therefore V = \frac{V}{B \times D} \tag{14}$$

Thus if we can meausuring the exact biomass and amount of DNA per unit biomass of an OTU, and divide the amount of DNA by this, we can exactly produce an amount of DNA equal to the abundance of the species, and concentration cancels out in the equation so does not need to be considered. However, these measurements are obviously impractical due to large numbers of species, and unlikely to produce good data, as PCR is unlikely to work well with low copy numbers.

Instead, let us assume the density of DNA D is a constant y, which allows us to treat OTUs solely by their biomass. Let us also apply a constant multiple z to our correction of M across all OTUs to give multiple DNA copies of each OTU in the pool. We shall encompass both of these constants in x, where $x = \frac{z}{y}$ and $z > y$. Thus, from equations 8 and 12:

$$M' = x \times \frac{M}{B} = M \times \frac{x}{B} = xA \tag{15}$$

$$\therefore V' = V \times \frac{x}{B} \tag{16}$$

This is a more simplified, practical version of these equations. Now, take the four size classes used in this study (Small, MeDium, Large, eXtralarge), noting that the mean

biomass of each are four times that of the next smallest class. Thus, taking the biomass off the Small class to be b

$$B_S = b \tag{17}$$
$$B_D = 4b \tag{18}$$
$$B_L = 4 \times B_M = 16b \tag{19}$$
$$B_X = 4 \times B_L = 64b \tag{20}$$

Applying this to equation 15 and simplifying, we get:

$$M'_S = M_S \times \frac{x}{b} \tag{21}$$
$$M'_D = M_D \times \frac{x}{4b} = \frac{M_D}{4} \times \frac{x}{b} \tag{22}$$
$$M'_L = M_L \times \frac{x}{16b} = \frac{M_L}{16} \times \frac{x}{b} \tag{23}$$
$$M'_X = M_X \times \frac{x}{64b} = \frac{M_X}{64} \times \frac{x}{b} \tag{24}$$

Through simplification we can see that $\frac{x}{b}$ becomes a constant for all four corrections, thus can be ignored, and to control for biomass we can simply divide the amount of DNA for each class by the multiple of the biomass means. Exactly the same treatment can be applied to the volumes V of the samples, from equation 15:

$$V'_S = V_S \times \frac{x}{b}$$
$$V'_D = \frac{V_D}{4} \times \frac{x}{b}$$
$$V'_L = \frac{V_L}{16} \times \frac{x}{b}$$
$$V'_X = \frac{V_X}{64} \times \frac{x}{b}$$

Of course, there are other variables that will affect the probability of recovery, in particular the assumptions made here that DNA density is constant between OTUs and between size classes; also, that recovery of OTUs are solely due to amount of DNA and not any other properties such as primer bias. Nonetheless, this approach should control for biomass bias to some extent; although, the results in the main paper suggest that this extent is relatively minor.

1.2 Clustering validation

The custom perl script NAPcluster is used to cluster filtered and merged reads to OTUs and map the reads against these OTUs. NAPcluster can iterate over different clustering methods and similarity values in order to examine the effects of these paramets on OTU number and other properties of the resultant dataset. For this data, NAPcluster was run using USEARCH cluster_otus versions 7.0, 8.0 and 9.2, and swarm, with clustering parameters of 0–15 % similarity ('cluster radius') for USEARCH and 1–63 bp differences for swarm. These values can be directly compared as the length of all reads was fixed at 418 bp, thus $similarity = \frac{differences}{418} \times 100$ so 12 differences is equivalent to a similarity of 2.87.

Pool	Minimum number of reads	Number of classes comprising pool	Class number correction	Minimum rarefaction target	Rarefaction target	Rounded rarefaction target
SizeP1	31010	1	0.25	124040	4625.75	4625
SizeP2	35067	2	0.5	70134	9251.50	9250
SizeP3	24896	3	0.75	33194.67	13877.25	13875
SizeP4	18503	4	1	18503	18503	18500
Prop.	36122	4	1	36122	18503	18500

Table S1: Calculation of rarefaction levels for pools in the size dataset. For each of the 9 tray samples, the minimum number of reads recovered for each set of the same pool type was found. A rarefaction correction was calculated from the number of classes, and this was used to calculate the minimum number of reads the most complex pool would require in order for each pool to have sufficient reads to rarefy. The lowest value of these was taken to be the maximum read number for rarefaction, and a target rarefaction was calculated from this value and the class number correction factor. If this resulted in decimals, the number for the least-complex pool was rounded down and multiplied by the number of classes for each of the other pools. The reads for each set of the same type of pool were then rarefied using the appropriate rounded rarefaction target for that type of pool. This ensured that the recovery of any one OTU across pools was not affected by simply having more OTUs in more complex pools. An equivalent process was used in the taxonomy dataset (not shown).

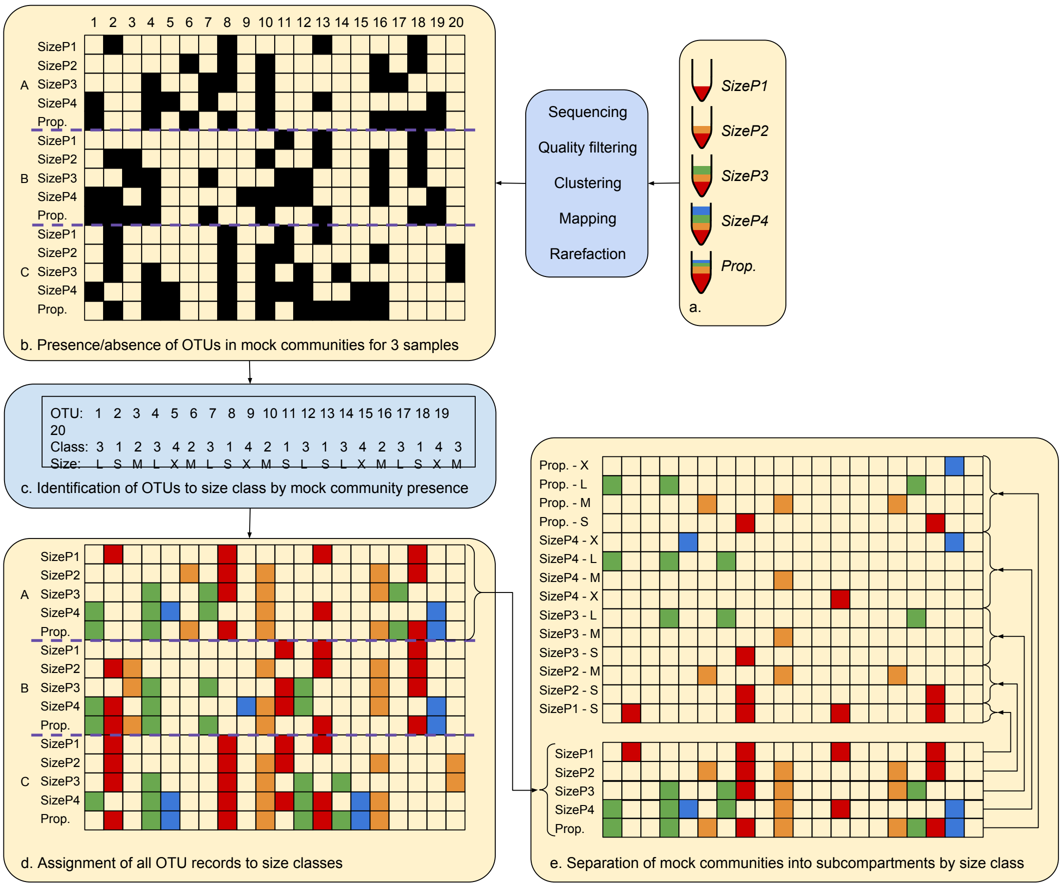

Figure S1: Data processing pipeline for separating composite pools into size class subcommunities. (a.) shows one of the nine mock community libraries as constructed in the lab (Figure 1 in main text). The subsequent sequence data was processed through a clustering pipeline (see text) to produce a table (b) of OTU (1-20) presence for each pool library within each tray sample (A-C, only three shown). Filled and open squares on the grid denote presence and absence respectively. For each OTU, the least complex pool in which it was present (across all tray samples) denoted its size class (c.). For example, OTU 2 was found in all types of pool, the least complex of which was SP1, which only contained small sizes. Therefore OTU 2 must have been a small species. OTU 6 was found in size classes SizeP2 and Prop., the least complex of which was SizeP2, which contained small and medium specimens. OTU 6 was not found in SizeP1 (small only), so this OTU must be a medium species. From this process, the size class composition of each pool library can be ascertained from its OTU composition (d). For further analysis, it was necessary to separate out the subcommunities comprising the OTUs of each size from each composite pool community. From tray samples (e.g. A) which had 5 mock communities, this generated 14 separate samples. The data from the 5 composite pool communites and/or the 14 subcommunities were used in subsequent analyses.

2 Supplementary Results

2.1 Clustering validation

Across all clustering methods and similarity levels (1–15 % for usearch or 0–63 differences for swarm), the number of OTUs ranged from 145 to 1800, with a mean of 739.4 (Supplementary Figure SS4). The range of iterations showed a clear effect of similarity level and clustering method (Supplementary Figure SS4), but with a distinct convergence around a similarity of 3 % for usearch, or 13 nucleotide differences for swarm (which is 3.1 % over 418 bp). All subsequent analyses of OTU distributions therefore were performed with OTU sets obtained with usearch version 9.2 setting a clustering radius of 3 % (similarity of 97 %).

2.2 Ordinations

Ordination based on beta dissimilarity (Figure SS3) clearly separated the different size based subcommunities (coloured points), which is unsurprising as these contain completely different OTUs. The position of the complete community (black points) reflects the medium, large and extra-large size classes relatively evenly, with the extent of turnover between each of these and the complete community roughly similar. However the complete community is considerably more similar to the smallest size class (red points). Within each size class, the two different construction methods (DNA-based and specimen-based) tend to cluster separately, while within these groupings subcommunities from the same tray sample tend to cluster more closely. The pattern of clustering is largely similar for both incidence-based and abundance-based beta diversity. Ordination of taxon subcommunities based on pairwise beta dissimilarity showed clear clusters based on taxon classes: similar to the size experiment, this is unsurprising as these contained completely different OTUs (Figure S3). Clustering patterns were very similar between incidence-based and abundance-based dissimilarity measures. Within taxon classes, subcommunities from the same tray sample (but different composite pools) tend to cluster together. The complete community was generally clustered distinctly from the taxon classes, disregarding the ‘complete’ TaxP1–P3 that consisted only of one taxon class each.

Class	Order	Number of sequences	Number forward matches	Number reverse matches	Number complete matches	Percent forward matches	Percent reverse matches	Percent complete matches
Arachnida	Amblypygi	3	3	3	3	100	100	100
Arachnida	Araneae	45	37	45	37	82	100	82
Arachnida	Astigmata	11	0	11	0	0	100	0
Arachnida	Ixodida	61	61	61	61	100	100	100
Arachnida	Mesostigmata	10	10	10	10	100	100	100
Arachnida	Opiliones	3	3	3	3	100	100	100
Arachnida	Oribatida	2	0	2	0	0	100	0
Arachnida	Pseudoscorpiones	4	4	4	4	100	100	100
Arachnida	Ricinulei	8	8	8	8	100	100	100
Arachnida	Scorpiones	9	8	8	8	89	89	89
Arachnida	Solifugae	3	3	3	3	100	100	100
Arachnida	Trombidiformes	36	10	36	10	28	100	28
Arachnida	Uropygi	1	1	1	1	100	100	100
Chilopoda	Geophilomorpha	2	2	2	2	100	100	100
Chilopoda	Lithobiomorpha	6	6	6	6	100	100	100
Chilopoda	Scutigeromorpha	2	2	2	2	100	100	100
Collembola	Cryptopygus	2	2	2	2	100	100	100
Collembola	Frieseinae	2	2	2	2	100	100	100
Collembola	Hypogastruridae	1	1	1	1	100	100	100
Collembola	Isotominae	2	2	2	2	100	100	100
Collembola	Onychiurinae	2	2	2	2	100	100	100
Collembola	Orchesellinae	2	2	2	2	100	100	100
Collembola	Paleonurini	2	2	2	2	100	100	100
Collembola	Poduridae	2	2	2	2	100	100	100
Collembola	Sminthuridae	2	2	2	2	100	100	100
Collembola	Tetrodontophorinae	1	1	1	1	100	100	100
Diplopoda	Callipodida	2	2	2	2	100	100	100
Diplopoda	Julida	2	2	2	2	100	100	100
Diplopoda	Playtdesmida	2	2	2	2	100	100	100
Diplopoda	Sphaerotheriida	2	2	2	2	100	100	100
Diplopoda	Spirobolida	2	2	2	2	100	100	100
Diplopoda	Spirostreptida	2	2	2	2	100	100	100
Diplura	Diplura	14	14	14	14	100	100	100
Insecta	Archaeognatha	10	10	10	10	100	100	100
Insecta	Blattodea	56	56	56	56	100	100	100
Insecta	Coleoptera	139	139	139	139	100	100	100
Insecta	Dermaptera	1	1	1	1	100	100	100
Insecta	Diptera	290	289	289	289	100	100	100
Insecta	Ephemeroptera	3	3	3	3	100	100	100
Insecta	Hemiptera	162	149	162	149	92	100	92
Insecta	Hymenoptera	65	38	65	38	58	100	58
Insecta	Lepidoptera	408	408	402	402	100	99	99
Insecta	Mantodea	4	4	4	4	100	100	100
Insecta	Mantophasmatodea	2	2	2	2	100	100	100
Insecta	Mecoptera	5	5	5	5	100	100	100
Insecta	Megaloptera	19	19	18	18	100	95	95
Insecta	Neuroptera	26	26	26	26	100	100	100
Insecta	Odonata	24	22	24	22	92	100	92
Insecta	Orthoptera	156	151	149	149	97	96	96
Insecta	Phasmatodea	19	19	19	19	100	100	100
Insecta	Phthiraptera	12	6	12	6	50	100	50
Insecta	Plecoptera	12	12	12	12	100	100	100
Insecta	Psocoptera	9	9	9	9	100	100	100
Insecta	Raphidioptera	2	2	2	2	100	100	100
Insecta	Siphonaptera	2	2	2	2	100	100	100
Insecta	Strepsiptera	2	0	2	0	0	100	0
Insecta	Thysanoptera	8	6	8	6	75	100	75
Insecta	Trichoptera	2	2	2	2	100	100	100
Insecta	Zoraptera	2	2	2	2	100	100	100
Insecta	Zygentoma	5	5	5	5	100	100	100
Malacostraca	Isopoda	6	6	6	6	100	100	100
Protura	Protura	6	4	6	4	67	100	67
No hits	No hits	6	6	6	6	100	100	100

Table S2: Table detailing the results of mapping the primers Ill_B_F and fol_degen_rev to full-length CO1 sequences of arthropods downloaded from GenBank. The number of sequences column reports the number of sequences in each family downloaded from GenBank. The subsequent sets of columns report the number and percentage of total sequences onto which the forward, the reverse and both forward and reverse primers successfully matched.

Dataset	Sample type	Set	Number of communities	β-diversity
Size	Size class	Small	40	0.9658
		Medium	31	0.9652
		Large	22	0.9567
		Extra-large	13	0.9637
	Composite size pool	SizeP1	9	0.9234
		SizeP2	9	0.9178
		SizeP3	9	0.9218
		SizeP4	9	0.9225
		Prop.	9	0.8328
Taxonomy	Taxon class	Coleoptera	28	0.9484
		Formicidae	32	0.9606
		Acari	32	0.9614
		Araneae	20	0.9301
		Hymenoptera	16	0.9329
		Hemiptera	12	0.8569
		Diptera	8	0.8925
		Collembola	4	0.7000
	Composite taxon pool	TaxP1	4	0.9135
		TaxP2	4	0.8919
		TaxP3	4	0.9241
		TaxP4	4	0.9008
		TaxP5	4	0.9109
		TaxP6	4	0.8635
		TaxP7	4	0.8703
		TaxP8	4	0.8332
		TaxP9	4	0.8633
		TaxP10	4	0.8647

Table S3: Table detailing Jaccard beta-diversity measures between samples within sample types. Sample types refer to groupings of size- or taxon-based subcommunities, or the size or taxon composite pools. For example, the small set refers to beta-diversity among all small subcommunities identified from the different size composite pools; the TaxP6 set refers to beta-diversity among the TaxP6 composite pools

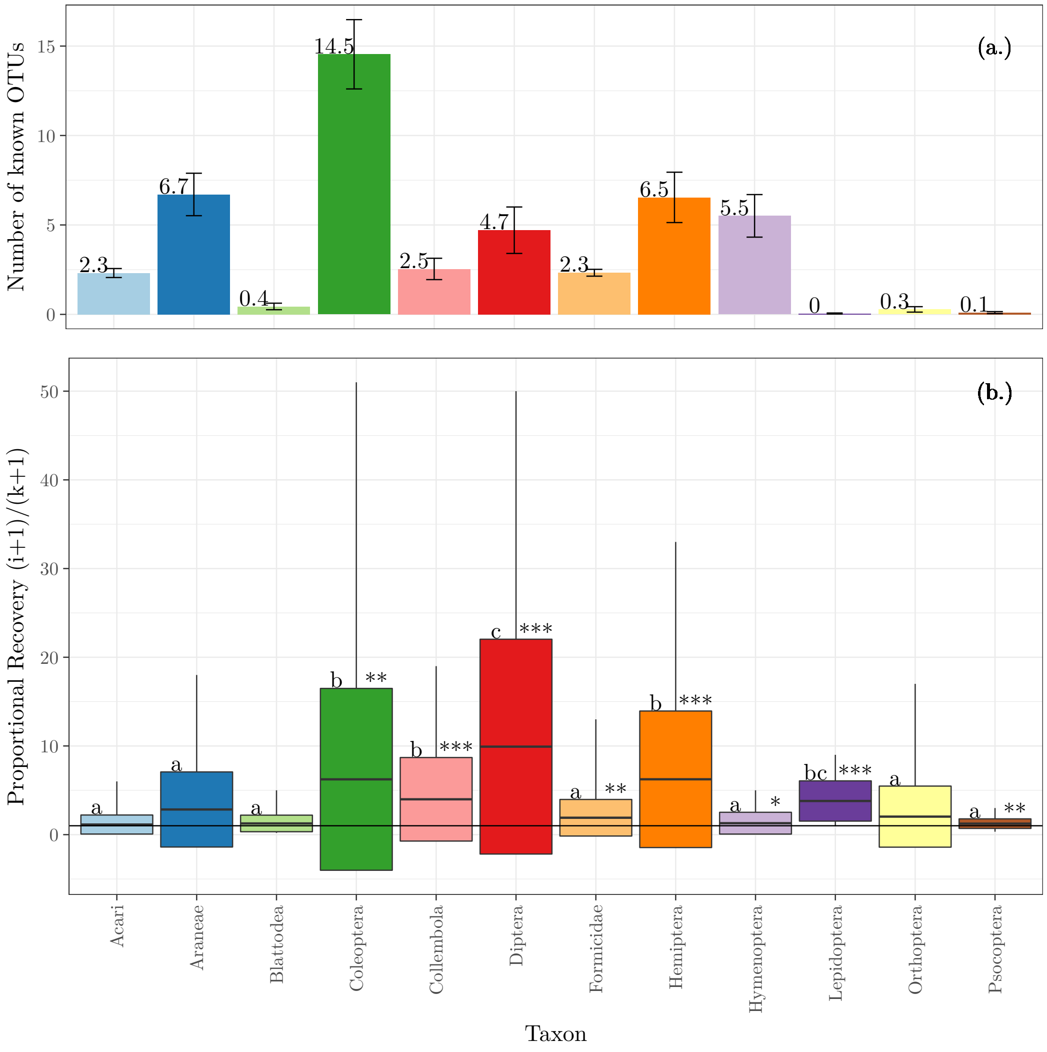

Figure S2: (a.) bar plot showing mean (numbers) and standard error of the number of known morphospecies of each taxon per sample for the set of samples for which morphospecies sorting was carried out. (b.) boxplot showing proportional recovery of taxa. Boxplots show mean and one standard deviation, with whiskers showing minimum and maximum values. Proportional recovery is calculated $\frac{i+1}{k+1}$ where i is the number of OTUs recovered for a taxon and k is the number of morphospecies in a taxon. Horizontal line denotes a proportional recovery of 1, i.e. where number of OTUs = number of morphospecies. Letters denote significant differences between taxa, stars denote a significant difference from 1 (* $0.01 < p < 0.05$, ** $0.001 < p < 0.01$, *** $p < 0.001$).

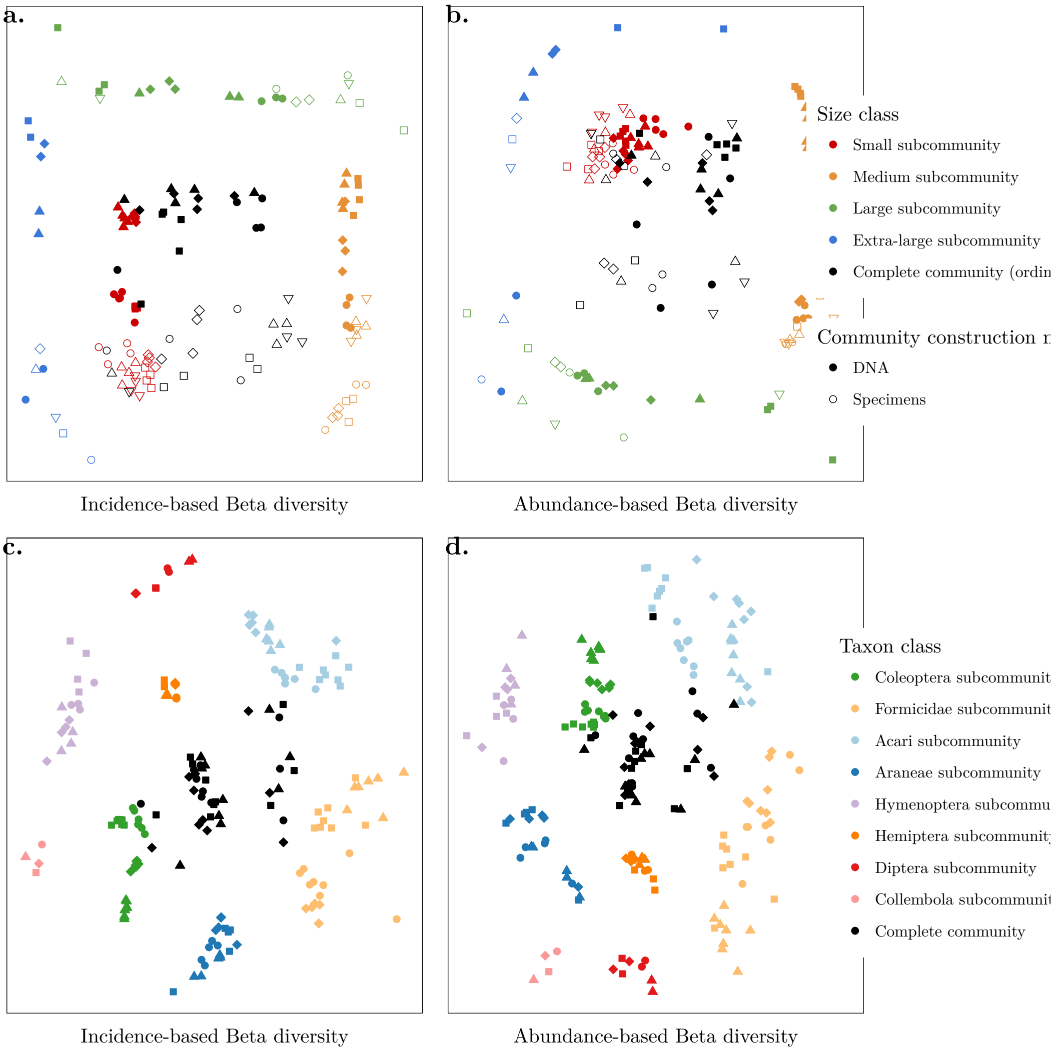

Figure S3: NMDS ordinations of compositional dissimilarity in size pool composite communities (black) and class subcommunities (colours; top: size, bottom: taxon). Shapes refer to the original tray sample, and in top plots only, filled and open points refer to method of experimental community construction. Left ordinations display data calculated using presence-absence data only (Jaccard dissimilarity) and right plots using read numbers (Bray-Curtis dissimilarity). For example, in the top figure, a red filled square point would be the small size class subcommunity from the ”square” tray constructed using separate DNA extracts from different size classes; a black square filled point would be a composite pool community from the same original tray. In the bottom figure, an orange triangular point would be the Hemiptera subcommunity from the ”triangle” tray; a black circular point labelled would be a composite pool community from the ”circle” tray. Axes are square-root transformed to better observe differences between the experimental communities.

Dataset	Pool	Samples	Reads per pool		OTUs per pool	
			Mean pre-rarefaction	Post-rarefaction	Mean pre-rarefaction	Mean post-rarefaction
Size	SizeP1	9	43453	4625	93	67
	SizeP2	9	46356	9250	123	94
	SizeP3	9	42588	13875	124	106
	SizeP4	9	38649	18500	133	120
	Prop.	4	45902	18500	209	192
	Total unique OTUs across size dataset				714	650
Taxonomy	TaxP1	4	27064	2234	61	38
	TaxP2	4	43194	2234	39	14
	TaxP3	4	46517	2234	42	13
	TaxP4	4	41195	4468	43	21
	TaxP5	4	27586	6702	98	52
	TaxP6	4	22303	8936	87	73
	TaxP7	4	27614	11170	98	86
	TaxP8	4	23728	13404	132	120
	TaxP9	4	22725	15638	151	136
	TaxP10	4	31923	17872	155	138
	Total unique OTUs across taxonomy dataset				551	471

Table S4: Table detailing the results of rarefaction of the two datasets. Rows show the different pool types, columns show the total number of tray samples for which these pool types were constructed, the mean reads per pool recovered post-clustering and the number of reads per pool after rarefaction. The mean number of OTUs these reads represent both before and after rarefaction are shown. Note that OTUs that were represented by only a single read post-clustering were removed. The total unique OTUs across the entire dataset is also reported.

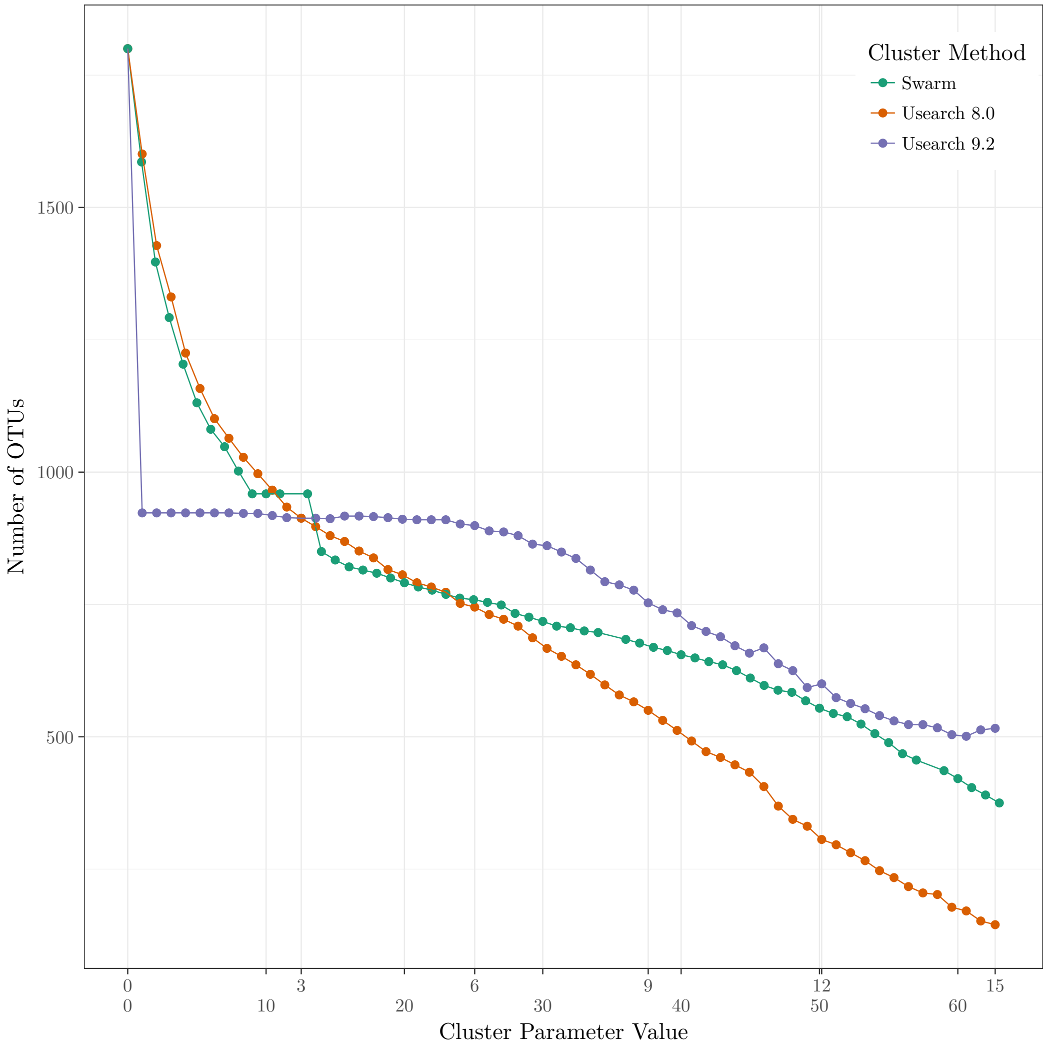

Figure S4: The number of OTUs generated by three different clustering methods over a range of "Cluster Parameter Values", i.e. similarity threshold for usearch 8.0 and usearch 9.2 (top values on x axis) and number of difference for swarm (bottom values on x axis). Coloured points and lines show the three different cluster methods. All methods used the same strict pre-clustering parameters: any reads not 418 bp were rejected, and unique sequences with fewer than 5 copies were also discarded. Remaining reads were denoised and chimera filtered before clustering.
